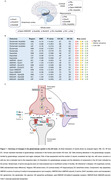# The Glutamatergic System in Alzheimer’s Disease: A Systematic Review with Meta‐analysis

**DOI:** 10.1002/alz.093255

**Published:** 2025-01-03

**Authors:** Carolina Soares, Lucas Uglione Da Ros, Luiza Santos Machado, Andreia Silva da Rocha, Gabriela Lazzarotto, Giovanna Carello‐Collar, Marco De Bastiani, João Pedro Ferrari‐Souza, Firoza Z Lussier, Diogo O. Souza, Pedro Rosa‐Neto, Tharick A. Pascoal, Eduardo R. Zimmer

**Affiliations:** ^1^ University of Pittsburgh, Pittsburgh, PA USA; ^2^ UFRGS, Porto Alegre, RS Brazil; ^3^ Universidade Federal Do Rio Grade Do Sul, Porto Alegre, Rio Grande do Sul Brazil; ^4^ Universidade Federal do Rio Grande do Sul, Porto Alegre, Rio Grande do Sul Brazil; ^5^ Federal University of Rio Grande do Sul, Porto Alegre Brazil; ^6^ UFRGS, Porto Alegre Brazil; ^7^ Universidade Federal do Rio Grande do Sul, Porto Alegre, RS Brazil; ^8^ Universidade Federal do Rio Grande do Sul, Porto Alegre Brazil; ^9^ Translational Neuroimaging Laboratory, The McGill University Research Centre for Studies in Aging, Montréal, QC Canada

## Abstract

**Background:**

Glutamatergic neurotransmission system dysregulation may play an important role in the pathophysiology of Alzheimer’s disease (AD). However, reported results on glutamatergic components across brain regions are contradictory. Here, we conducted a systematic review with meta‐analysis to examine whether there are consistent glutamatergic abnormalities in the human AD brain.

**Method:**

We searched PubMed and Web of Science(database origin‐October 2023) reports evaluating glutamate, glutamine, glutaminase, glutamine synthetase, glutamate reuptake, aspartate, excitatory amino acid transporters, vesicular glutamate transporters, glycine, D‐serine, metabotropic and ionotropic glutamate receptors in the AD human brain. The studies were synthesized by outcome and brain region. We included cortical regions, the whole brain(cortical and subcortical regions combined), the entorhinal cortex and the hippocampus. Pooled effect sizes were determined with standardized mean differences (SMD), random effects adjusted by false discovery rate, and heterogeneity was examined by I^2^statistics.

**Result:**

The search retrieved 6 936 articles,63 meeting the inclusion criteria(N = 709CN/786AD;mean age 75/79). We showed that the brain of AD individuals presents decreased glutamate (SMD = ‐0.82;P<0.001) and aspartate levels (SMD = ‐0.64; I^2^ = 89.71%; P = 0.006), and reuptake (SMD = ‐0.75;P<0.001). We also found reduced AMPAR‐GluA2/3 levels(SMD = ‐0.63;P = 0.046), hypofunctional NMDAR (SMD = ‐0.60;P<0.001) and selective reduction of NMDAR‐GluN2B subunit levels(SMD = ‐1.07;P<0.001). Regional differences include lower glutamate levels in cortical areas and aspartate levels in cortical areas and in the hippocampus, reduced glutamate reuptake, reduced AMPAR‐GluA2/3 in the entorhinal cortex, hypofunction of NMDAR in cortical areas, and a decrease in NMDAR‐GluN2B subunit levels in the entorhinal cortex and hippocampus. Other parameters studied were not altered.

**Conclusion:**

Our findings show depletion of the glutamatergic system and emphasize the importance of understanding glutamate‐mediated neurotoxicity in AD. This study has implications for the development of therapies and biomarkers in AD.